# FXR Controls the Tumor Suppressor NDRG2 and FXR Agonists Reduce Liver Tumor Growth and Metastasis in an Orthotopic Mouse Xenograft Model

**DOI:** 10.1371/journal.pone.0043044

**Published:** 2012-10-09

**Authors:** Ulrich Deuschle, Julia Schüler, Andreas Schulz, Thomas Schlüter, Olaf Kinzel, Ulrich Abel, Claus Kremoser

**Affiliations:** 1 Department of Research, Phenex Pharmaceuticals AG, Heidelberg, Germany; 2 Department of In Vivo Tumor Biology, Oncotest GmbH, Freiburg, Germany; 3 Department of Medicinal Chemistry, Merz Pharmaceuticals GmbH, Frankfurt, Germany; University of Hong Kong, Hong Kong

## Abstract

The farnesoid X receptor (FXR) is expressed predominantly in tissues exposed to high levels of bile acids and controls bile acid and lipid homeostasis. FXR^−/−^ mice develop hepatocellular carcinoma (HCC) and show an increased prevalence for intestinal malignancies, suggesting a role of FXR as a tumor suppressor in enterohepatic tissues. The N-myc downstream-regulated gene 2 (NDRG2) has been recognized as a tumor suppressor gene, which is downregulated in human hepatocellular carcinoma, colorectal carcinoma and many other malignancies.

We show reduced NDRG2 mRNA in livers of FXR^−/−^ mice compared to wild type mice and both, FXR and NDRG2 mRNAs, are reduced in human HCC compared to normal liver. Gene reporter assays and Chromatin Immunoprecipitation data support that FXR directly controls NDRG2 transcription via IR1-type element(s) identified in the first introns of the human, mouse and rat NDRG2 genes. NDRG2 mRNA was induced by non-steroidal FXR agonists in livers of mice and the magnitude of induction of NDRG2 mRNA in three different human hepatoma cell lines was increased when ectopically expressing human FXR. Growth and metastasis of SK-Hep-1 cells was strongly reduced by non-steroidal FXR agonists in an orthotopic liver xenograft tumor model. Ectopic expression of FXR in SK-Hep1 cells reduced tumor growth and metastasis potential of corresponding cells and increased the anti-tumor efficacy of FXR agonists, which may be partly mediated via increased NDRG2 expression. FXR agonists may show a potential in the prevention and/or treatment of human hepatocellular carcinoma, a devastating malignancy with increasing prevalence and limited therapeutic options.

## Introduction

The Farnesoid X Receptor (FXR, NR1H4) is a member of the nuclear hormone receptor superfamily, predominantly expressed in tissues exposed to high levels of bile acids, such as the entire gastrointestinal tract, the liver, the bile duct and gallbladder. FXR mRNA can also be detected in tissues such as the adrenals, kidneys and adipose tissues [1], [2]. FXR senses bile acids (such as Chenodeoxycholic acid, CDCA) as endogenous ligands, is a master regulator of bile acid homeostasis and prevents bile acid–induced liver toxicity by regulating directly and indirectly (e.g. via Small Heterodimer Partner, SHP, NR0B2) the expression of numerous genes involved in bile acid synthesis, conjugation, and transport [3]–[7]. Activation of FXR by synthetic derivatives of the natural bile acid ligands, such as 6-Ethyl-Chenodeoxycholic Acid (6-ECDCA), or by synthetic non-steroidal agonists like GW4064 [8], results in beneficial metabolic adjustments in different mouse models such as glucose lowering, insulin sensitisation, triglyceride and cholesterol lowering [9]–[11]. Moreover, activation of FXR results in hepatoprotection in mouse models of Non Alcoholic Fatty Liver Disease (NAFLD) possibly mediated via a reduction of lipid accumulation, fibrosis and inflammation [12]–[14].

FXR^−/−^ mice spontaneously develop hepatocellular carcinoma beyond 12 months of age, suggesting that FXR has a prominent function as a tumor suppressor against liver tumor formation [15], [16] but also against intestinal tumor formation [17], [18], [19]. Of direct clinical importance is the tumor-stage dependent reduction of both FXR mRNA and FXR protein in human colon carcinoma [20], [21].

Using genome-wide Chromatin Immunoprecipitation followed by sequencing (ChIP-Seq), two groups have identified numerous genes containing *in vivo* FXR binding sites in liver and intestine [22], [23]. A limited number of those genes that are controlled via FXR may be particularly relevant for the tumor-protective activity of FXR. The orphan receptor small heterodimer partner SHP (NR0B2), is transcriptionally up-regulated as a direct target gene of FXR in the mouse liver and is involved in a negative feed-back regulation of bile acid synthesis via repression of Cyp7a1 transcription [3], [4]. SHP^−/−^ mice do also spontaneously develop liver tumors beyond 12 months of age, similar to what is found in FXR^−/−^ mice [15], [16], suggesting a tumor suppressing activity of SHP in mouse liver [24]. Of clinical significance is the epigenetic silencing of the SHP gene in human liver tumor isolates and established HCC-derived cell lines [25]. Interestingly, adenovirus mediated expression of SHP in HepG2 cells does reduce their tumor growth rate in nude mice compared to HepG2 cells carrying a control adenovirus [25]. This suggests that SHP may be among such gene products controlled by FXR that contribute to the tumor suppressing activity of FXR.

N-myc downstream regulated gene 2 (NDRG2) was recently reported as a candidate tumor suppressor in human liver cancer metastasis and it is transcriptionally reduced in HCC [26]. Furthermore, reduced NDRG2 expression was published in high-risk adenoma, colorectal carcinoma [27]–[30], glioblastoma [31] thyroid cancer [32], esophageal cancer [33] renal cancer [34], gallbladder carcinoma [35] and breast cancer [36].

Here we show that NDRG2 is a direct transcriptional target of FXR in mouse liver and human hepatoma cell lines. We further demonstrate that Ndrg2 mRNA is reduced in livers of FXR^−/−^ mice compared to wild type mice and Ndrg2 mRNA can be induced by non-steroidal FXR agonists in livers of wild type mice. In humans, both, NDRG2 and FXR mRNA's are reduced in primary hepatocellular carcinoma samples of different tumor stages compared to normal human liver or non-HCC liver diseased human liver. Finally, we provide evidence that stable over-expression of FXR in SK-Hep-1 human hepatoma cells does reduce proliferation and migration of these cells in an FXR agonist dependent fashion *in vitro* and tumor growth and metastasis in an orthotopic xenograft mouse model *in vivo*.

## Results

### NDRG2 is a novel direct target gene of FXR in mouse liver and human hepatoma cell lines

**Figure 1:**
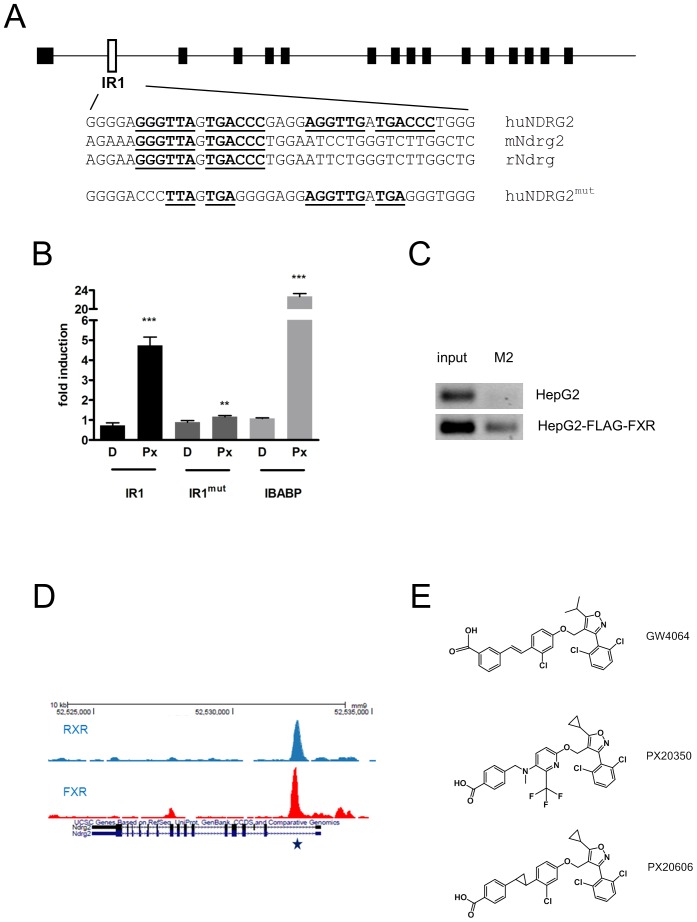
Identification of functional IR1 site(s) in NDRG2. **A**, Schematic representation of the NDRG2 gene with the IR1 site(s) located in the first intron. Human, mouse and rat intronic sequences with IR1 elements (in bold) are shown below (IR1 elements start at positions +1560, +757 and +1394 respectively). A version of the human NDRG2 response element with mutated IR1 sites is shown below. **B**, Luciferase reporter assays from HEK 293 cells co-transfected with pTReX-Dest30-hFXRα2 and a luciferase reporter plasmid containing either one copy of the wild type IR1 element (IR1) or the mutated element (IR1^mut^) from the human NDRG2 gene or 2 copies of the human IBABP-RE (IBABP). The fold induction in presence of 0.5 µM Px20350 (Px) versus DMSO control (D) of firefly luciferase activities (normalized against renilla luciferase) of triplicate experiments is shown. **C**, Amplification of a 196 bp DNA fragment encompassing the IR1 sequences from chromatin prepared by ChIP from HepG2 or HepG2-FLAG-FXR cells. PCR reactions from input DNA and DNA derived from the immunoprecipitation with the M2 anti-FLAG antibody are indicated **D**, Localization of murine FXRα and RXRα by ChIP-seq to a site corresponding to the predicted IR1 recognition element (star) in the first intron of the mouse Ndrg2 gene on chromosome 14 in mouse liver. The FXRα and RXRα UCSC genome browser tracks shown for the NDRG2 gene were retrieved from genome wide ChIP-seq datasets published by Thomas et al. [22] and Boergesen et al. [41], respectively. **E**, Chemical structures of FXR agonists. Among different structural changes, the unfavourable stilbene linker present in GW4064 was replaced by an amino-methylene linker in Px20350 [40]. The further modified Px20606 [53] yielded improved *in vivo* pharmakokinetic properties compared to Px20350. A characterization of FXR agonists in *in-vitro* assays is presented in Table 1.

In order to identify novel genes that might be controlled by FXR, we employed a bioinformatics-driven approach that relied on the identification of putative FXR response elements of the IR1-type around transcriptional start sites of known annotated human, mouse and rat genes. First, genomic DNA sequences from human, mouse and rat genes flanking the first exons of all annotated genes in the region from −20,000 bp to +10,000 bp were extracted into separate databases. These sequence stretches were subsequently searched for the occurrence of IR1-type recognition sequences corresponding to the consensus [GA]GGT[TC]A-N-T[AG]ACC[TC] that is known to be recognized by FXR in identified target genes [37], [38], [39]. Numerous genes in human, mouse and rat, containing at least one such IR1 consensus like sequence were identified in this way (428, 483 and 506 genes respectively). Genes containing such IR1 consensus sequences at comparable positions in all three species were considered good candidates for transcriptional control by FXR. Among those genes, NDRG2 harbours a conserved IR1 sequence (GGGTTAGTGACCC) within the first intron in all three species (Fig. 1A) and gene expression data suggest co-expression of FXR and NDRG2 mRNA's in human and mouse liver (http://biogps.org). Interestingly, there is a second IR1-like sequence (AGGTTGATGACCC) just downstream of the first sequence in the human NDRG2 gene, which is absent in the mouse and rat genes (Fig. 1A). The DNA fragment containing the IR1-type sequences from the human NDRG2 gene (see Fig. 1A) was cloned into a luciferase reporter plasmid and tested for function in transient co-transfection experiments together with an FXR expression plasmid in HEK293 cells, which do not express endogenous FXR. The wild type NDRG2 IR1 sequence (Fig. 1A) conferred inducibility of the corresponding luciferase reporter to the FXR agonist PX20350 (Fig. 1B, Fig. 1E, [40]) while a mutated version of the IR1 type sequence (see Fig. 1A) conferred only marginal inducibility (Fig. 1B). A luciferase reporter plasmid with a tandem repeat of the FXR response element from the ileal bile acid binding protein promoter was also tested for comparison (IBABP, Fig. 1B). The inducibility by PX20350 was strictly dependent on co-transfection of the FXR expression plasmid, suggesting that FXR is able to functionally recognize the IR1 sequence(s) derived from the first intron of human NDRG2 *in vitro*. By chromatin immunoprecipitation with HepG2 cells and a HepG2 derivative ectopically expressing a FLAG-tagged human FXR (HepG2-FLAG-FXR), we could amplify a 196 bp DNA fragment by PCR, encompassing the predicted IR1 (Fig. 1A) sequences only from chromatin immunoprecipitated with the M2 anti-FLAG antibody in HepG2 cells ectopically expressing FLAG-tagged FXR and not from HepG2 cells (Fig. 1C). Using ChIP-seq, Thomas and colleagues [38] identified binding sites of murine FXRα on DNA extracted from livers of wild type mice in a genome wide fashion. Within this publically available dataset (http://genome.ucsc.edu/cgi-bin/hgGateway), we identified a strong FXRα binding signal that was located right on the predicted IR1 sequence in the first intron of mouse Ndrg2 (Fig. 1D). A similar FXRα binding event was also identified within the data from a published ChIP-Seq study with mouse liver, recently published by Chong and colleagues (data not shown, [39]). ChIP-Seq data from another experiment with mouse liver revealed that also RXRα is localized to this site *in vivo* (Fig. 1D, RXRα track provided by Dr. H.G. Stunnenberg, [41]). This suggests that a FXRα/RXRα heterodimer may be bound at this site *in vivo*.

From these data we conclude that FXR can bind to the intronic IR1 sequences in mouse liver *in vivo* and in human HepG2 cells *in vitro*.

**Figure 2:**
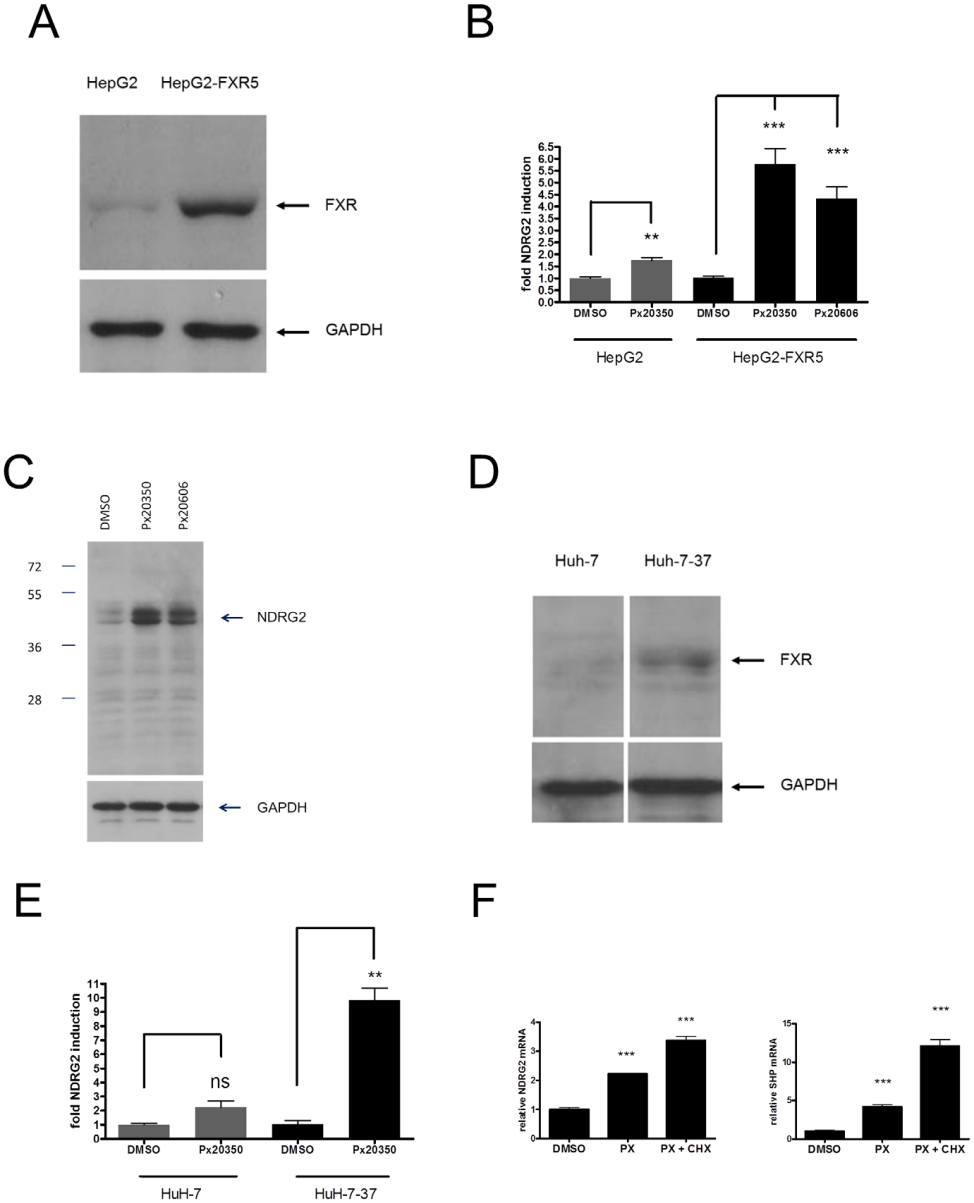
Induction of NDRG2 in human hepatoma cell lines. **A**, Ectopic expression of FXR in HepG2-FXR5 compared to HepG2 parental cells. Positions of bands representing FXR and GAPDH proteins in the western blots from aliquots of whole cell extracts are indicated. **B**, Dose dependent induction of NDRG2 mRNA in HepG2 and HepG2-FXR5 cells by the FXR agonist PX20350. HepG2 or HepG2-FXR5 cells were grown for 18 h in medium containing either DMSO, 0.5 µM of PX20350 or 0.5 µM of PX20606 in 96 well plates as indicated and relative fold induction of NDRG2 mRNA over the DMSO control was determined by RT-qPCR using the ΔΔCt method. **C**, Induction of NDRG2 protein by the FXR agonists Px20350 and PX20606 in HepG2-FXR5 cells. HepG2-FXR5 cells were grown to a density of around 60% and further cultivated for 18 h in medium supplemented with either DMSO as a vehicle, 0.5 µM PX20350 or 0.5 µM PX20606 as indicated and aliquots of whole cell extracts analysed by western blots. Relative positions of size marker protein bands and the position of NDRG2 and GAPDH proteins are indicated. **D**, Stable overproduction of FXR in HuH-7-37 compared to HuH-7 cells. Positions of bands representing FXR and GAPDH proteins analysed by western blots from aliquots of whole cell extracts are indicated. **E**, Dose dependent induction of NDRG2 mRNA in HuH7 and HuH7-37 cells by FXR agonist Px20350. HuH7 or HuH7-37 cells were grown for 18 h in medium containing either DMSO or increasing concentrations of PX20350 in DMSO in 96 well plates and relative fold induction over DMSO control of NDRG2 mRNA was determined by RT- qPCR using the ΔΔCt method. **F**, NDRG2 is a direct transcriptional target of FXR. HepG2-FXR5 cells, grown in quadruplicates in 12 well plates at a density of around 60%, were further incubated for 4 hours in growth medium supplemented with either DMSO, 0.5 µM PX20350 (PX) or 0.5 µM PX20350 together with 10 µM Cycloheximide (CHX). The relative fold induction of NDRG2- and SHP- mRNAs over DMSO as control were determined by RT- qPCR using the ΔΔCt method and a Student's t-test was performed (*** = p<0.001).

Next we generated human hepatoma cell lines by stable transfection of HepG2 and HuH-7 cells with a human FXR expression construct and selected for stable lines overexpressing human FXR (named HepG2-FXR5 and HuH-7-37; Fig. 2A and Fig. 2D). In both parental cell lines, NDRG2 mRNA was induced about two-fold by presence of 0.5 µM PX20350 in the growth medium for 18 hours when compared to the DMSO control. Ectopic overexpression of FXR did increase the amplitude of induction of NDRG2 mRNA (Fig. 2B) and NDRG2 protein (Fig. 2C) in HepG2-FXR5 cells and NDRG2 mRNA in HuH-7-37 cells (Fig. 2E). The observed double band for NDRG2 proteins in Fig. 2C may correspond either to the two NDRG2 isoforms (http://www.ncbi.nlm.nih.gov/gene/57447) and/or to differentially phosphorylated forms. The structurally related FXR agonist PX20606 (Fig. 1E) induced NDRG2 in HepG2 and HepG2-FXR5 cells comparable to PX20350 (Fig. 2B and Fig. 2C). On basis of its improved pharmacokinetic properties, PX20606 was chosen as a preferred FXR agonist for mouse *in vivo* experiments. The activities of the FXR agonists PX20350 and PX20606 in direct comparison to GW4064 [8] in biochemical and cellular assays are shown in Table 1.

**Table 1:** Activities of the different FXR agonists in different assay systems.

A				
FRET	mFXR		hFXR	
	EC_50_ [nM]	Efficacy [%]	EC_50_ [nM]	Efficacy [%]
GW4064	40	100	30	100
PX20606	1000	100	150	100
PX20350	83	95	10	110

B				
Gal4	mFXR		hFXR	
	EC_50_ [nM]	Efficacy [%]	EC_50_ [nM]	Efficacy [%]
GW4064	120	100	30	100
PX20606	220	160	50	95
PX20350	50	110	12	60

(**A**) Activities in the biochemical TR-FRET assay using the human FXR-LBD. (**B**) Activities in Gal4-FXR-LBD fusion cellular reporter assays using mouse (mFXR) and human FXR-LBDs (hFXR). Efficacy is provided as % relative to the maximum effect of GW4064 in the respective assays.

To further confirm that NDRG2 is a direct transcriptional target gene of FXR, we performed induction experiments with PX20350 in the absence or presence of 10 µM Cycloheximide (CHX), an inhibitor of protein synthesis. Similar to the established direct FXR target gene SHP, NDRG2 mRNA induction was not reduced by the protein synthesis inhibitor Cycloheximide, suggesting that NDRG2 is a direct target gene of FXR (Fig. 2F). Interestingly, both FXR target genes were induced even more strongly in the presence of Cycloheximide, a phenomenon deserving future investigation. In addition, an increase of NDRG2 mRNA in HepG2-FXR5 cells could be detected as early as 30 min after addition of PX20350 (data not shown), further supporting the view that NDRG2 is a direct transcriptional target of FXR. Similar results were obtained with HuH-7 and HuH-7-37 cells (data not shown).

### Ndrg2 is controlled by FXR in mouse liver

**Figure 3:**
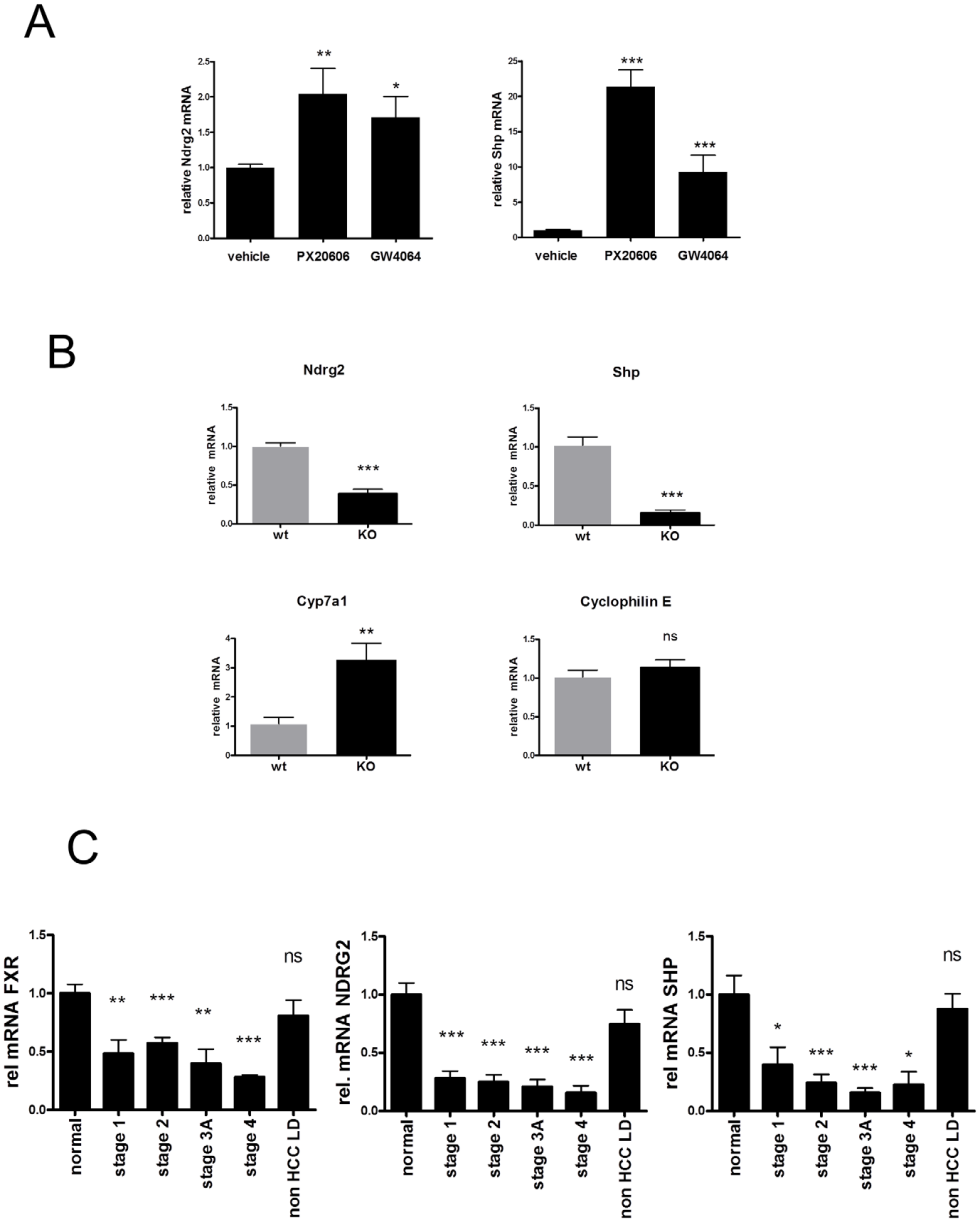
Ndrg2 mRNA is induced in wt mice by FXR agonists, reduced in livers of FXR^−/−^ versus wt mice and both FXR and NDRG2 mRNAs are reduced in human HCC versus normal liver. **A**, Ndrg2 mRNA induction in livers of C57Bl6 mice by FXR agonists. C57Bl6 mice on normal chow received a bolus of vehicle (n = 9), or 30 mg/kg of Px20606 or 30 mg/kg of GW4064 in vehicle (n = 6 each) by oral gavage. Mice were sacrificed after 4 h and liver RNA isolated for RT-qPCR analysis. Ndrg2 and Shp mRNA expression levels were calculated according to the ΔΔCt method using normalization to Tbp as a house keeping transcript and the mean level of Ndrg2 and Shp expression in vehicle treated mice were set to 1.0. Data are presented as the mean ± standard error of the mean (±SEM). Statistical significance in a nonpaired Student's t-test: *, ** and *** = p<0.05, <0.01 and <0.001. **B**, Quantitative determination of mRNA's in livers of wild type (wt) and FXK^−/−^ (KO) mice. Relative mRNA levels of Ndrg2, Shp, Cyp7a1 and Cyclophilin E were determined by RT-qPCR from wt (n = 5) and FXR^−/−^ (n = 5) mice as indicated. Tbp-normalized relative mRNA levels of wt mice were set to 1.0 respectively. **C**, Decreased expression of FXR and the FXR target genes NDRG2 and SHP in human HCC. FXR, NDRG2 and SHP mRNA levels were quantified by RT- qPCR in 8 normal, 34 HCC from different stages (7 samples stage I, 8 samples of stages II and IIIA, 3 samples of stage IV) and 12 non-HCC liver disease (LD) derived cDNA's. Relative folds of mRNA expression levels between normal livers and the respective stages of HCC or non-HCC liver disease were calculated according to the ΔΔCt method using normalization to TBP. The mean levels of FXR, NDRG2 and SHP expression in normal livers were set to 1.0 respectively and relative mRNA levels are expressed as mean ± SEM. Statistical significance in a nonpaired Student's t-test: *, P<0.05; **, P<0.01; ***, P<0.001.

In order to address if Ndrg2 is controlled by FXR in mouse liver, we analysed the expression levels of Ndrg2 mRNA in livers of wild type mice 4 hours after oral gavage of 30 mg/kg of either PX20350 (Fig. 1E, [40]) or the FXR agonist GW4064 (Fig. 1E, [8]) compared to vehicle. Oral gavage of both FXR agonists did result in a significant induction of Ndrg2 mRNA in the livers of respective mice, as shown in Fig. 3A. The relative fold induction of Ndrg2 is lower than the induction of Shp (Fig. 3A). However, the absolute level of Ndrg2 mRNA in mouse liver is much higher compared to the Shp mRNA (around 16-fold), implicating an impressive increase in the absolute copy number of newly synthesized Ndrg2 transcripts upon FXR activation.

If Ndrg2 is directly controlled by FXR, loss of FXR expression in FXR^−/−^ mice might result in reduced expression of Ndrg2 mRNA in the liver. Quantitative Real Time PCR (RT-qPCR) indicated a significant reduction of Ndrg2 mRNA in livers of FXR^−/−^ mice compared to livers of wild type mice (Fig. 3B). Similarly, the level of Shp mRNA was decreased in livers of FXR^−/−^ mice. In contrast, Cyp7a1 mRNA, which is under negative feedback control via the FXR-SHP pathway, was increased and Cyclophilin E mRNA was unchanged, as expected (Fig. 3B).

### Coordinate reduction of FXR, NDRG2 and SHP in HCC versus normal liver

The data so far suggest that NDRG2 is a direct transcriptional target gene of FXR and that a reduction or loss of FXR expression correlates with a reduction of NDRG2 expression.

Such a reduction of NDRG2 mRNA has been reported in tumor tissue compared to adjacent “normal” tissue of patients with hepatocellular carcinoma [26]. We therefore analyzed the expression of genes including FXR and NDRG2 in a commercially available cDNA panel derived from liver specimens from healthy individuals and liver specimens from patients with stage I, II, IIIA and IV hepatocellular carcinoma as well as non-malignant liver diseases. As shown in Fig. 3C, the expression of FXR, NDRG2 and SHP mRNAs are significantly reduced in all tumor stages when compared to “normal” and to liver tissue from non-malignant liver disease. The expression of housekeeping genes, such as TBP, was found unchanged while the expected downregulation of KAI1 (CD82), as previously described [42], was confirmed in the liver tumor samples from this liver cDNA panel (data not shown).

Furthermore, publically available data from a genome wide gene expression study with matched “normal” and “tumor” tissues from 10 patients with HCC [43] were retrieved and analyzed for FXR and NDRG2 mRNA levels. Again, the mRNA levels of both, FXR and NDRG2, were found to be reduced significantly in tumor tissue compared to normal liver tissue (data not shown).

### FXR agonists reduce growth and metastasis potential of human SK-Hep-1 hepatoma cells *in vitro* dependent on FXR expression level and possibly via control of NDRG2

**Figure 4:**
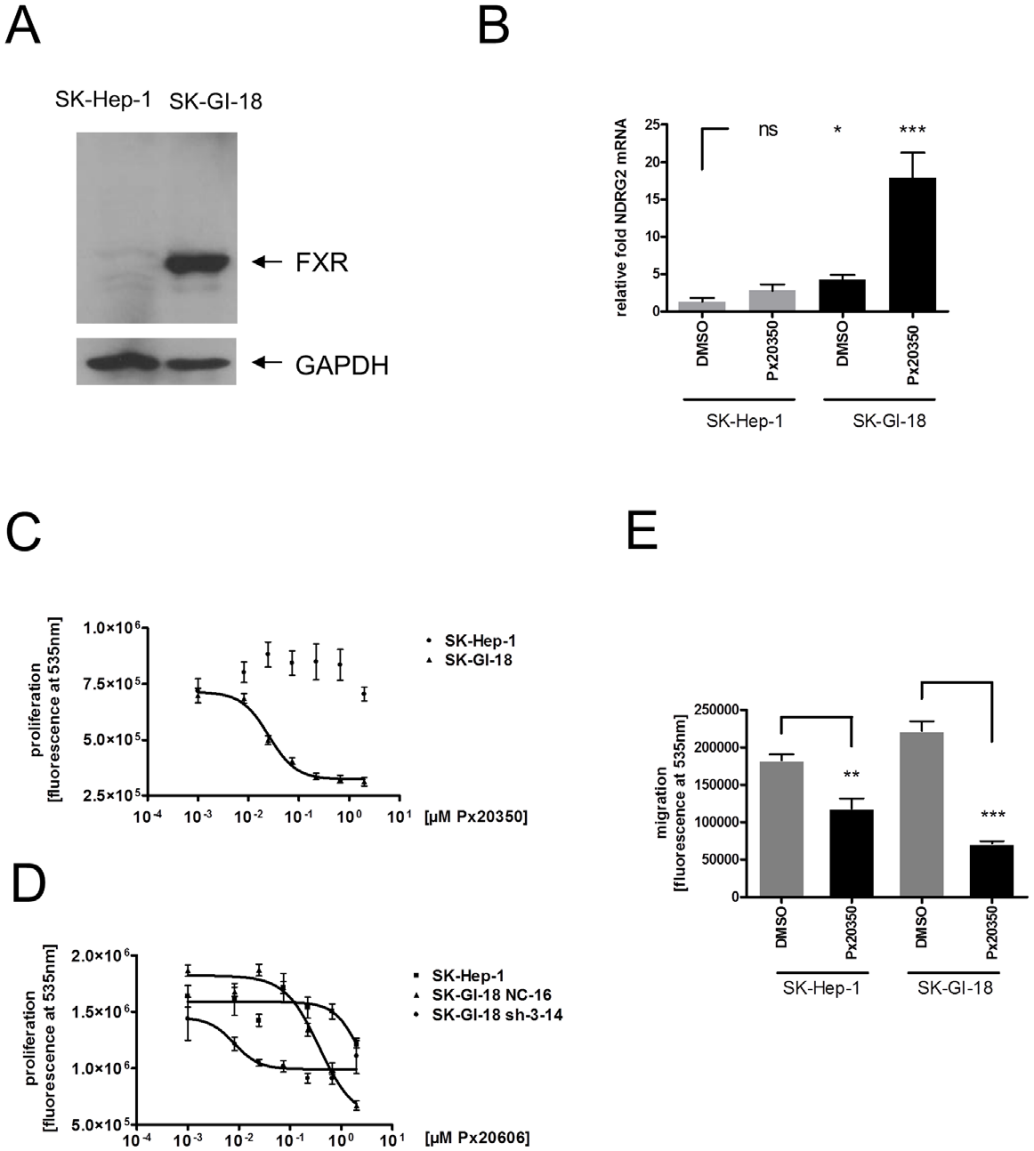
*In vitro* characterization of SK-Hep-1 and SK-GI-18 cells. **A**, Expression of FXR in SK-Hep-1 and the FXR overexpressing SK-GI-18 cells. The signals in western blot analysis from aliquots of whole cell extracts representing FXR and GAPDH are indicated. **B**, Increased expression and inducibility of NDRG2 mRNA by Px20350 in SK-GI-18 cells compared to parental SK-Hep-1 cells. SK-Hep-1 cells and SK-GI-18 cells were grown in 24-well plates (n = 4) in presence of DMSO or 0.5 µM Px20350 for 72 hours. Total RNA was isolated from cells at around 60% confluency. The diagram shows RT-qPCR data for NDRG2 mRNA drawn as relative fold increase over the TBP corrected values for DMSO treated SK-Hep-1 cells using the ΔΔCt method. Statistical significance in a nonpaired Student's t-test: *, ** and *** = p<0.05, <0.01 and <0.001. **C**, Influence of PX20350 on proliferation of SK-Hep-1 and SK-GI-18 cells *in vitro*. SK-Hep-1 or SK-GI-18 cells were seeded in a 96 well plate and grown for 4 days in presence of DMSO or increasing concentrations of FXR ligand Px20350. Relative proliferation was measured by flurescence determination of DNA content using the CyQuant Direct Proliferation assay. D. Inhibition of proliferation by PX20606 is blunted in SK-GI-18-sh3-14 cells stably expressing shRNA directed against NDRG2. SK-Hep-1, SK-GI-18 NC-16 expressing a negative control shRNA and SK-GI-18-3-14, expressing an NDRG2 shRNA were seeded at 3000 cells/well in a 96 well plate and grown for 3 days in presence of DMSO or increasing concentrations of PX20606. Relative proliferation was measured by flurescence determination of DNA content using the CyQuant Direct Proliferation assay. **E**, Migration of SK-GI-18 and SK-Hep-1 cells in presence or absence of Px20350 (1 µM). Cells were seeded at 7000 cells per 96 well (in quadruplicates) of a Oris Fibronectin Coated Plate (Platyplus) and seeding stoppers removed after 14 h and cells grown in medium with DMSO as a vehicle or 1 µM Px20350 in DMSO for another 72 hours before quantification of migrated cells using the Cyquant Direct Cell Proliferation Assay.

NDRG2 has been demonstrated to be a candidate suppressor of liver cancer and metastasis [26] and in particular, stable overexpression of NDRG2 was shown to suppress the invasion and migration of the highly invasive hepatoma cell line SK-Hep-1 [44] in an experimental xenograft model in the mouse [26]. We built on this finding and created a stable SK-Hep-1 derivative, named SK-GI-18, which ectopically expresses human FXR (variant FXRα2, NM_005123) under the control of the strong CMV promoter. While FXR protein was merely detected in the parental cell line SK-Hep-1, FXR protein was readily detectable in SK-GI-18 by Western Blot analysis (Fig. 4A). In SK-Hep-1 cells, the amount and inducibility of NDRG2 mRNA was comparably low while the relative amount and inducibility of NDRG2 mRNA was significantly increased in the SK-GI-18 cell line (Fig. 4B). Both cell lines do show similar growth rates under standard *in vitro* cell culture conditions. While the proliferation of parental SK-Hep-1 cells did not change strongly in presence of increasing concentrations of the FXR agonist PX20350, a significant PX20350 dose-dependent reduction in the rate of proliferation with the FXR expressing SK-GI-18 cells was observed *in vitro* (Fig. 4C, [40]). This dose dependent reduction in proliferation rate with the SK-GI-18 cells could not be explained by cytotoxicity or apoptosis-inducing activity of PX20350 at the concentrations used. When testing both cell lines in a migration assay, both showed a decrease of migration activity in presence of 0.5 µM Px20350 in the medium compared to the DMSO control. The extent of inhibition of migration was, however, much stronger with the FXR overexpressing cell line SK-GI-18 compared to SK-Hep-1 (Fig. 4E).

In order to address if NDRG2 does participate in mediating the anti-proliferative effect of FXR agonists on the SK-GI-18 cells, we stably transfected SK-GI-18 cells with shRNA silencing vectors in which small hairpin sequences directed against NDRG2 are expressed under control of the human U1 promoter. Stabile clones with four different small hairpin constructs and a negative control sequence were stably established by selection with 250 µg/ml Hygromycin B and tested for the expression of FXR and NDRG2 mRNAs in presence or absence of the FXR agonist PX20606. Clones with the shRNA Nr.3 (see Material and Methods) showed robust reduction of NDRG2 in presence and absence of PX20606 compared to the negative control small hairpin (reduction of around 85%, Supporting Fig. S1). The proliferation of one selected clone (SK-GI-18-sh-3-14) showed a blunted reduction in proliferation upon addition of increasing concentrations of the FXR agonist PX20606 when compared to the SK-GI-18-sh-NC-16 clone expressing a negative control small hairpin (Fig. 4D, Material and Methods). Again, SK-Hep-1 cells, only marginally expressing FXR, were barely influenced by increasing amounts of PX20606. This result suggests, that the effects of FXR activation on the reduction of proliferation of SK-GI-18 cells may at least partially be mediated via upregulation of NDRG2. It will be interesting to identify additional gene products controlled by FXR that do contribute to the observed phenotype.

### FXR and FXR agonists exert anti-tumorigenic activities in an orthotopic xenograft model in nude mice

The cell line SK-GI-18 (Fig. 4) was selected from more than 10 stable SK-Hep-1 derivatives originally generated on basis of the stability of the FXR-overexpression phenotype in absence of any selective pressure (G418 was used at 1000 µg/ml for initial selection of stable transfectants but subsequently omitted) after subcloning by limited dilution in *in vitro* cell culture. The SK-GI-18 cell line was chosen accordingly for testing the effects of FXR agonists in an orthotopic tumor model in female nude mice established at Oncotest GmbH in Freiburg, Germany.

Female nude mice were injected with either 5×10^6^ SK-Hep-1 or 5×10^6^ SK-GI-18 cells into one liver lobe, respectively. For quantitative detection of tumor load, fluorophore-coupled anti-human CD10 antibodies were injected (i.v.) 3 days after implantation and fluorescence was determined using the *In-Vivo* Imaging System FX from Kodak on the following day. X-ray photographs were taken in parallel to allow for both quantification (*in vivo* fluorescence imaging) and localization (X-ray) of the tumor cell load, at the primary sites of injection in the liver as well as at sites of metastasis (e.g. liver, gut, lymph nodes and bone). Animals were randomized on day 4 and subsequently treatment was started by daily oral gavage with vehicle or compounds suspended in vehicle. In a first pilot experiment with four animals per group, we observed a strongly reduced growth of tumors in the livers of mice that received SK-GI-18 cells, overexpressing FXR, compared to mice that received SK-Hep-1, during an observation period of 32 days (data not shown).

**Figure 5:**
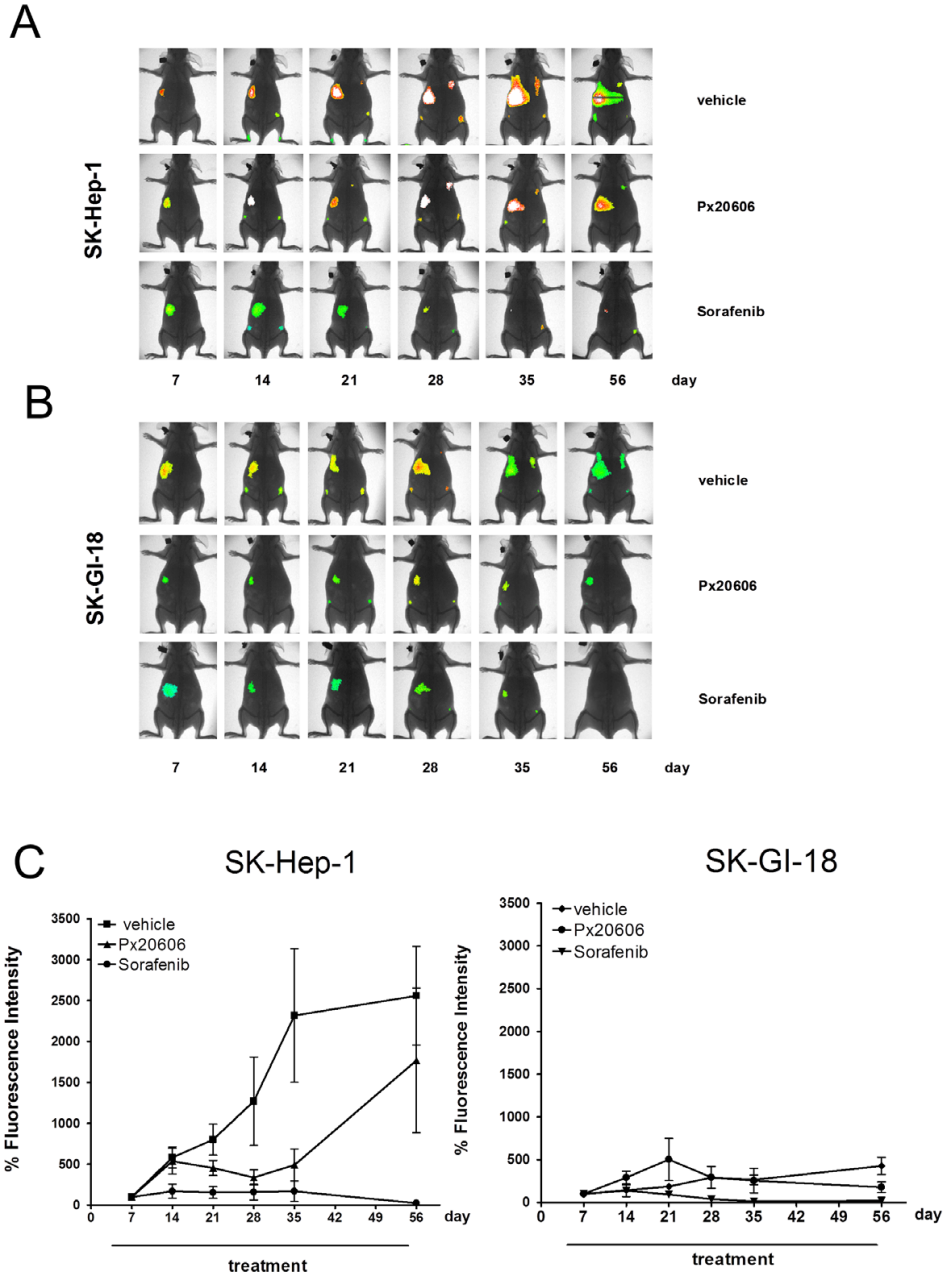
Time course of tumor growth and metastasis. **A and B**, Nude mice were inoculated with either 5×10^6^ SK-Hep-1 (A) or SK-GI-18 cells (B), randomized into groups and animals were treated by daily gavage starting on day 4 with vehicle, Px20606 (10 mg/kg/d) or Sorafenib (100 mg/kg/d) as indicated. *In vivo* imaging pictures for representative animals from each group that received SK-Hep-1 (A) or SK-GI-18 (B) cells at the indicated days are displayed. **C**, Time course of tumor development in the liver. The *in vivo* fluorescence intensities at day 7 were set to 100% for each group and data plotted for data gathered at days 7, 14, 21, 28, 35 and 56. The sharp rise of fluorescence in the SK-Hep-1/Px20606 group from day 35 to day 56 is due to sharp increases in tumor sizes in just three individual animals out of a total of nine animals (fluorescence data from these animals on day 56 are circled in Fig. 6A).

**Figure 6:**
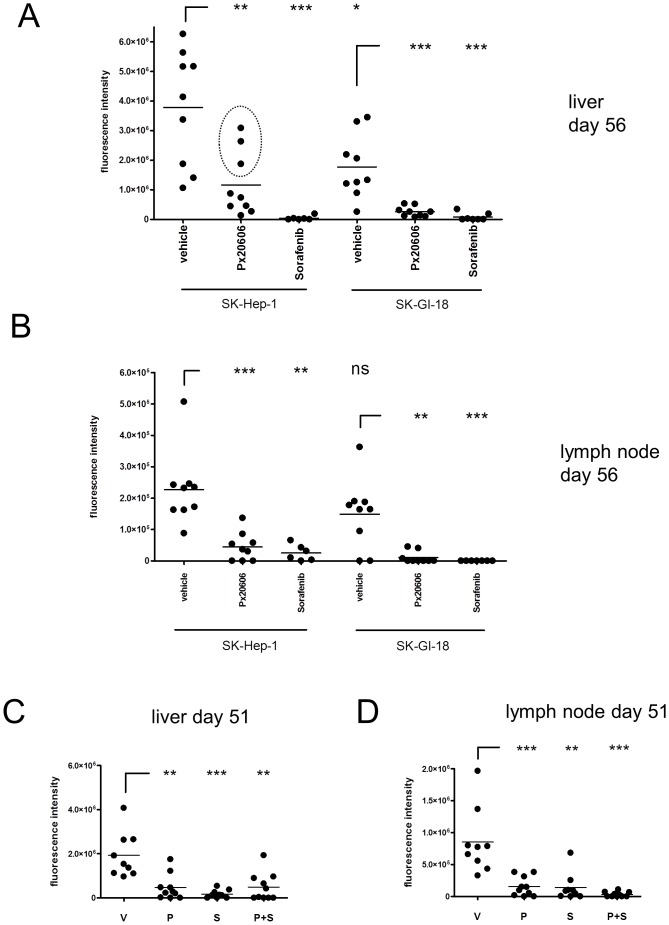
The effect of treatments on tumor cell growth in liver or lymph nodes. **A**, Tumor load in the liver (primary tumor) at the end of the experiment on day 56. Scatter plots of fluorescence intensities with horizontal bars as mean are shown. Group sizes at beginning of the experiments were 9 animals each, with the exception of SK-Hep-1/Sorafenib (n = 7) and SK-GI-18/Sorafenib (n = 8). For statistical analysis non-paired Student's t-tests were performed as indicated (ns = >0.05; * = <0.05; ** = <0.01; *** = <0.001). Three animals in the SK-Hep-1/Px20606 group showed a sharp increase in fluorescence from day 35 to day 56 and are highlighted by a circle. **B**, Lymphnode metastases at the end of the experiment on day 56. Scatter plots of fluorescence intensities localized to lymph nodes with horizontal bars as mean are shown. Group sizes at beginning were 9 animals each, with the exception of SK-Hep-1/Sorafenib (n = 7) and SK-GI-18/Sorafenib (n = 8). For statistical analysis non-paired Student's t-tests were performed as indicated (ns = >0.05; * = <0.05; ** = <0.01; *** = <0.001). **C**, Combination of PX20606 and Sorafenib does not further reduce growth of tumors derived from SK-GI-18 cells in the liver. Nude mice were injected with 5×10^6^ SK-GI-18 cells and *in vivo* imaging performed on day 4 before randomization of animals into the four treatment groups vehicle (V), 10 mg/kg/d PX20606 (P), Sorafenib 50 mg/kg/d (S) or 10 mg/kg/d PX20606+Sorafenib 50 mg/kg/d (P+S). Scatter plots of fluorescence intensities in the liver at termination of the experiment on day 51 with horizontal bars as mean are shown. Group sizes were 9 animals each for vehicle and Sorafenib and 10 animals each for PX20606 and PX20606+Sorafenib. For statistical analysis non-paired Student's t-tests against were performed as indicated (ns = >0.05; * = <0.05; ** = <0.01;*** = <0.001). **D**, Combination of PX20606 and Sorafenib further reduces metastasis into lymph nodes. The same animals as in Fig. 6C were analysed for lymph node metastases. Scatter plots of fluorescence intensities at sites of lymph nodes with horizontal bars as mean are shown. Group sizes were 9 animals each for vehicle and Sorafenib and 10 animals each for PX20606 and PX20606+Sorafenib. For statistical analysis non-paired Student's t-tests against were performed as indicated (ns = >0.05; * = <0.05; ** = <0.01; *** = <0.001).

In a second experiment, female nude mice received either 5×10^6^ SK-Hep-1 or 5×10^6^ SK-GI-18 cells by injection into one lobe of the liver, but group sizes were increased (7–9 animals). After 3 days of recovery, and starting with day 4, mice were daily gavaged with vehicle or 10 mg/kg/d of the FXR agonists PX20606 in vehicle or 100 mg/kg/d of Sorafenib (Nexavar™) in vehicle until the animals were finally sacrificed on day 56. For this experiment, PX20606 was chosen because of its improved pharmacokinetic properties compared to PX20350 (Fig. 1E). Sorafenib (Nexavar™), a multi-kinase inhibitor directed against a broad range of protein kinases (e.g. VEGFR, PDGFR, Raf) is the only medicament approved for the treatment of advanced hepatocellular cancer and advanced renal cell carcinoma to date and was used here as reference therapeutic treatment [48]. The individual tumor load was determined weekly by *in vivo* imaging until the end of the experiment, 56 days after injection of tumor cells. *In vivo* imaging data of representative animals from each group showing the respective fluorescence intensities overlayed on the X-ray pictures are presented in Fig. 5A and Fig. 5B. The *in vivo* fluorescence intensities, determined on day 7 within each group were set to 100% and *in vivo* fluorescence intensity data plotted for days 14, 21, 28, 35 and 56 in Fig. 5C. Compared to SK-Hep-1 cells, the SK-GI-18 cells ectopically expressing FXR, showed a strikingly reduced tumor growth in the liver. This could be the result of activation of the ectopically expressed FXR by endogenous bile acids in the liver. Oral gavage with PX20606 (10 mg/kg/d) or Sorafenib (100 mg/kg/d) reduced tumor growth in the liver derived from both, SK-Hep-1 and SK-GI-18 cells, when compared to the vehicle control (Fig. 5C). Notably, there was heterogeneity among the tumor growth rates within the animals of the SK-Hep-1/PX20606 group, namely just three individual animals showed a sharp increase of tumor growth from day 35 to day 56 (circled in Fig. 6A). The basis for this heterogeneity phenomenon will have to be investigated more thoroughly in the future.

Fluorescence intensity data on day 56 are quantitatively represented in the scatter plots shown in Fig. 6A and Fig. 6B. Within the experimental limitations of this model, animals from all groups showed comparable tumor burden at the primary site of hepatoma cell injection on day 7 of the experiment (data not shown). Treatment regimes, which started on day 4, did obviously not yet had a strong impact on tumor burden at day 7 compared to vehicle control (Fig. 5C). Both, the SK-Hep-1 but also the SK-GI-18 cells when gavaged with vehicle only, did continuously grow at the primary sites of tumor cell injection until the end of the experiment on day 56 (Fig. 5C and Fig. 6A). First lymph node metastases were readily visible starting on day 14 (Fig. 5A and 5B).

Both, SK-Hep-1 and SK-GI-18 derived tumors showed a significantly reduced growth rate upon treatment with the FXR agonist PX20606. Without the non-steroidal FXR agonist, however, there was a strikingly reduced growth of the SK-GI-18 derived tumors compared to SK-Hep-1 derived tumors in the liver (Fig. 5C and Fig. 6A). This could be the result of activation of ectopically expressed FXR by endogenous FXR agonists (e.g. bile acids such as CDCA).

Very interestingly, however, SK-GI-18 derived metastases in lymph nodes did not grow significantly less aggressive than those derived from SK-Hep-1 cells (Fig. 5C and Fig. 6B). This might be explained by lower levels of endogenous bile acids in lymph nodes compared to the liver, resulting in reduced FXR activation in the periphery.

Oral gavage of Sorafenib (100 mg/kg/d) did, as expected, significantly reduce the growth of tumors in the liver and metastases in lymph nodes derived from both SK-Hep-1 and SK-GI-18 cells when compared to vehicle treatment (Fig. 6A and Fig. 6B). The synthetic FXR agonist, PX20606, did also significantly reduce the growth of liver tumors and metastases in lymph nodes derived from SK-Hep-1 and SK-GI-18 cells compared to vehicle treated animals (Fig. 6A and Fig. 6B). Interestingly, Sorafenib treatment completely prevented growth of metastases in lymph nodes derived from SK-GI-18 cells (Table 2A). Metastases in the bone were detected on day 56 in 4 out of 9 mice transplanted with SK-Hep-1 cells and receiving vehicle. Treatment with Px20606 or Sorafenib did completely interfere with formation of bone metastases (Table 2B). Importantly, mice receiving the SK-GI-18 cells overexpressing FXR did not show any bone metastases, again supporting the view that the metastasis potential of SK-Hep-1 is generally reduced by ectopic expression of FXR in the SK-GI-18 cells, even in the absence of PX20606 mediated FXR stimulation (Table 2B).

**Table 2:** Number of animals with metastases in lymph nodes (A) and bone (B) and the total numbers of animals in each group surviving till day 56 (in parentheses) are shown.

A. Lymph node metastases			
Cells injected	Vehicle	Px20606	Sorafenib
SK-Hep-1	9 (9)	6 (9)	5 (6)
SK-GI-18	7 (9)	4 (9)	0 (7)

B. Bone metastases			
Cells injected	Vehicle	Px20606	Sorafenib
SK-Hep-1	4 (9)	0 (9)	1 (6)
SK-GI-18	0 (9)	0 (9)	0 (7)

One animal died in each of the groups receiving Sorafenib, in the course of the experiment.

These data suggest that Sorafenib might be particularly effective in preventing metastasis to lymph nodes with hepatoma cells ectopically expressing FXR.

Accordingly, in a separate experiment, SK-GI-18 cells were orthotopically implanted into nude mice and daily gavaged with vehicle, PX20606 (10 mg/kg/d), Sorafenib (50 mg/kg/d) or a combination of Px20606 and Sorafenib (10 and 50 mg/kg/d respectively) starting on day 4 until the end of this experiment on day 51. Tumor load (fluorescence intensity) in the liver was again significantly reduced by treatment with PX20606 (P), Sorafenib (S) and the combination of Px20606 and Sorafenib (P+S) as shown in Fig. 6C. Sorafenib seemed slightly more effective than PX20606 and the combination (P+S) was directly comparable to PX20606 alone (Fig. 6C). The combination of PX20606 with Sorafenib seemed not to be superior to the individual treatment regimes in the liver. On the contrary, the reduction of metastases in the lymph nodes was apparently more pronounced in the combination treatment (P+S, Fig.6D).

## Discussion

Herein we show that the bile acid receptor FXR, most likely as a heterodimer with RXR, controls transcription of NDRG2 though IR1 response elements identified in the first introns of mouse, human and rat NDRG2 genes, respectively. We show that FXR, when activated by a non-steroidal agonist, induced the transcription of the liver tumor suppressor candidate NDRG2 [26] in mouse liver and three different human hepatoma cell lines. Ndrg2 and Shp mRNAs were significantly reduced in livers of FXR^−/−^ mice compared to livers from wild type mice. It is therefore tempting to speculate, that the downregulation of Ndrg2 mRNA in FXR^−/−^ mice might contribute to the time dependent development of hepatocellular carcinoma in these mice. This hypothesis could be directly tested in the future by liver specific deletion of NDRG2 in adult mice or shRNA-mediated knock-down of NDRG2. However, loss of FXR also results in downregulation of SHP, and SHP^−/−^ mice also do develop HCC in a time-dependent fashion [24]. Moreover, since FXR controls many hepatoprotective genes, including genes involved in detoxification of reactive oxygen species [14], loss of NDRG2 is more likely just one of the factors mediated by loss of FXR function that contribute to the development of HCC. We show here, that both FXR and NDRG2 mRNA levels are significantly downregulated in different stages of human HCC compared to “normal” liver, corroborating recently published work [26], [28], [45]. Neither FXR nor NDRG2 seemed significantly down-regulated in the combined set of 13 tested non-HCC liver-disease cDNA samples originating from patients with liver cirrhosis (n = 5), fatty liver (n = 5) and chronic hepatitis (n = 3) compared to “normal” liver (Fig. 3C). However, the data suggest a trend towards slightly reduced levels of FXR and NDRG2 mRNA's in these liver diseases and in particular, the three samples from patients with chronic hepatitis showed a statistically significant reduction of both FXR and NDRG2 mRNAs compared to livers from normal subjects. This will have to be carefully investigated in the future with larger collections of samples from patients with chronic hepatitis and in particular carriers of Hepatitis B and Hepatitis C viruses, which are at an increased risk of developing HCC [49]. Very recently, reduction of FXR expression was demonstrated in cholangiocarcinoma and biliary tract carcinoma [46], [47] and in tumor versus normal liver tissue from HCC patients [51], [52] and NDRG2 expression was found downregulated in gallbladder carcinoma with poor outcome [35]. In addition, a recent report showed a tumor-stage dependent reduction of both FXR mRNA and FXR protein in human colon carcinoma [20], [21]. Future experiments will have to address if NDRG2 is directly controlled by FXR in tissues of the biliary tract including gall bladder and colon and whether FXR mediated loss of NDRG2 expression might be involved in tumorigenesis in these organs as well.

We could demonstrate that ectopic expression of FXR in the SK-Hep-1 derivative SK-GI-18 resulted in an increase in basal and FXR agonist-induced expression of NDRG2 and in an FXR agonist-dependent reduction in growth rate and migration potential *in vitro*. We also showed that interference with NDRG2 expression by small hairpin RNA expression blunted the FXR agonist dependent reduction in proliferation *in vitro*, suggesting that NDRG2 might mediate at least to some extent the growth reducing effects of FXR agonists in the respective cell lines. We employed an orthotopic xenograft tumor model in nude mice combined with *in vivo* imaging to follow growth of hepatoma cell-derived tumors at the primary site of injection in the liver and metastasis into lymph nodes, gut and bone marrow. The growth of tumors derived from FXR over-expressing SK-GI-18 cells was significantly reduced in the liver and in the gut compared to tumors derived from the parental SK-Hep-1 cells. In contrast, there was no significant difference in metastasis of SK-GI-18 cells into lymph nodes compared to the parental SK-Hep-1 cells. After oral application of the strong FXR agonist PX20606, however, both, growth of tumors in the liver and metastases in the lymph nodes were significantly reduced both compared to vehicle as well as compared to SK-Hep-1. This might be explained by a higher availability/activity of the non-steroidal FXR agonist Px20606 at these peripheral sites compared to the endogenous bile acids which are more concentrated in enterohepatic tissues. The growth and metastasis suppressing activity of FXR agonist Px20606 (10 mg/kg/d) in this orthotopic xenograft model with either SK-Hep-1 or SK-GI-18 is only slightly less efficacious compared to Sorafenib (Nexavar™), presently the only medicament approved for treatment of advanced HCC [48], which was administered at a much higher dose, however (100 mg/kg/d or 50 mg/kg/d). These results are strikingly reminiscent to findings of Lee and colleagues, showing that ectopic expression of NDRG2 in SK-Hep-1 cells did reduce tumor growth and metastasis of SK-Hep-1 derived tumors in a mouse xenograft tumor model [26].

Interestingly, Sorafenib showed a higher efficacy to interfere with metastasis of SK-GI-18 cells to lymph nodes compared to the parental SK-Hep-1 cells. Moreover, the combined treatment with Sorafenib (50 mg/kg/d) and Px20606 (10 mg/kg/d) did further reduce the metastasis of SK-GI-18 cells to lymph nodes in the orthotopic xenograft model. These findings suggest that pathways activated by FXR agonists and inhibited by the pan-tyrosine kinase inhibitor Sorafenib may synergize to interfere with effective metastasis of hepatoma cells. Future studies will have to address in detail the mechanism for this synergistic activity on tumor metastasis.

In view of our results and previously published studies we propose that FXR functionally acts as a tumor suppressor in liver and presumably other enterohepatic tissues. We propose that reduction of FXR expression or FXR function over an extended time period could lead to a reduced expression of direct and indirect target genes, including NDRG2 and SHP, mediating the anti-tumorigenic functions of FXR. NDRG2 has been implicated as a tumor suppressor in a number of malignancies [26]–[36], but the molecular mechanisms still have to be explored in more detail. However, NDRG2 was shown to restrain the Wnt/TCF/beta-catenin signalling pathway and NDRG2 decreases in a tumor-stage dependent fashion in colorectal carcinoma [28]. On the contrary, activated Wnt/beta-cathenin pathway is found very frequently in HCC [50] and an increased beta-catenin pathway activity was also found in spontaneous hepatocellular carcinoma in FXR^−/−^ mice [45]. We want to point out that the FXR agonist PX20606 is effective in reducing tumor growth in liver and metastasis to lymph nodes of tumors derived from implanted SK-Hep-1 cells, which barely do express FXR. We therefore propose that the tumor-suppressive activity of FXR might be mediated in part via activation of endogenous FXR in mouse cells, namely liver parenchymal, endothelial or even lymphoid cells (e.g. Kupffer cells), resulting in a changed microenvironment unfavourable for tumor growth or metastasis. We are presently generating FXR^−/−^ mice on a nude background in order to directly test the involvement of mouse FXR on the observed behaviour. More advanced stages of human HCC seem to go along with progressive de-differentiation of malignant liver cells and progressively reduced FXR expression and/or activity. However, residual FXR expression in malignant liver cells and FXR expression/activity in non-malignant surrounding tissue could both represent targets for antitumor activities of FXR agonists. This may be the basis for novel medicines that could not only prevent or slow down the development of a primary tumor or progression of tumor growth at early stages but potentially also tumor growth or metastasis at later clinical stages of HCC. The absence of strong cytotoxicity of FXR agonists like PX20350 or PX20606 and their hepatoprotective activities may also make them possible candidates for a combination with classical chemotherapy or multi-kinase-targeted anti-tumor therapies such as Sorafenib.

## Materials and Methods

### Ethics Statement (Animal Health and Monitoring)

All animal experiments were conducted according to the guidelines of the German Animal Welfare Act (Tierschutzgesetz) and under the permission numbers of the Regierungspräsidium Freiburg, Germany G-10/05 and 35-9185.64/1. Animal health was examined prior to tumor implantation and randomization to ensure that only animals without any symptoms of disease were selected to enter testing procedures. During the experiments, animals were monitored twice daily regarding tumor burden, general condition, food and water supply. Crl:NU-*Foxn1^nu^* mice (nude mice) were purchased from Charles River, Erkrath, Germany and obtained in microisolators in barrier conditions. At 6–8 weeks of age, mice were injected with SK-HEP-1 or SK-GI-18 cells.

### Compounds and Reagents

GW4064, PX20350 and PX20606 were synthesized according to published synthesis protocols [8], and checked for identity and purity by ^1^H-NMR and LC-MS before being tested in FXR activity assays. All other chemicals used were of analytical grade or higher. Sorafenib (as tosylate salt) was derived from prescription strength Nexavar 200 mg tablets (Bayer Vital GmbH, Leverkusen, Germany). Compounds were dissolved in 100% DMSO (Sigma-Aldrich, Munich, Germany) and diluted in growth medium to a final concentration containing 0.2% DMSO for *in vitro* studies. DMSO was added to cultures at a concentration of 0.2% (v/v) as a vehicle control. For *in vivo* experiments in nude mice, compounds were suspended in 0.5% Polyvinylpyrrolidone K30 (BASF, Ludwigshafen, Germany), 0.1% Polysorbat 80 (Roth, Karlsruhe, Germany) in 150 mM NaCl (Sigma-Aldrich, München, Germany) as a vehicle and administered by oral gavage at 2.5 ml/kg dosing volume. Sorafenib (half of the daily dose) was gavaged twice a day with a 6 hour time lag.

For short term induction experiments (4 hours) in C57Bl/6 mice (see Fig. 3A), a different vehicle (emulsion of 75% corn oil and 25% water) was used to allow for faster absorption of the lipophilic FXR agonists. The CyQuant Direct Proliferation Assay was obtained from Life Technologies, Carlsbad, U.S.A.. The *Oris*™ Cell Migration Assay with Fibronectin coated 96 well plate was obtained from Platyplus Technologies, Madison, U.S.A.. Mouse monoclonal anti-FXR antibody (clone A9033A, IgG2a) was purchased from R&D Systems, Wiesbaden-Nordenstadt, Germany. Goat anti-NDRG2 antibodies (E-20) were purchased from Santa Cruz, Heidelberg, Germany. Mouse anti FLAG M2 antibodies (F1804), Mouse-anti GAPDH antibodies (G8795), goat anti-mouse IgG-Alkaline Phosphatase Conjugate (A3688) and rabbit anti-goat IgG-Alkaline Phosphatase Conjugate (A4062) were all obtained from Sigma-Aldrich, Munich, Germany and Lumi-Phos WB Chemiluminescent Substrate (34150) was obtained via Thermo Scientific, Bonn, Germany.

### Cell lines

Hep-G2 (ACC-180), SK-Hep-1 (ACC-141) and Hek-293 (ACC 305) cells were obtained from DSZM, Braunschweig, Germany. HuH-7 (JCRB0403) cells were obtained from the Health Science Research Resources Bank, Osaka, Japan. Cells were cultivated in RPMI-1640 (Hep-G2 and SK-Hep-1 cells), MEM (Hek-293) or DMEM (HuH-7) containing 8.6% fetal calf serum supplemented with 2 mM L-Alanyl-L-Glutamine (all purchased via Sigma-Aldrich, Munich, Germany).

### Determination of proliferation and migration of hepatoma cells *in vitro*

For *in vitro* proliferation cells were plated at 1000 cells per well of a 96 well plate and after 16 h, growth medium with different concentrations of the respective FXR agonist was added. After 72 hours, the DNA content was determined using a spectro-fluorometer (Envision, Perkin Elmer, Boston, U.S.A.) using the CyQuant Direct Proliferation Assay (according to the manufacturers recommendations.

For determination of *in vitro* migration, cells were plated at 7000 cells per well using the *Oris*™ Cell Migration Assay with Fibronectin coated 96 well plates according to the manufacturers manual of Platyplus Technologies, Madison, USA. Fluorescence was determined using a spectro-fluorometer (Envision, Perkin Elmer, Boston, U.S.A.).

### Generation of stable cell transfectants expressing human FXR

For overexpression of human FXR (isoform α2, NM_005123), the coding region was amplified using Gateway compatible primers GGGGACAAGTTTGTACAAAAAAGCAGGC-TCG**ATGGGATCAAAAATGAATCTC** and GGGGACCACTTTGTACAAGAAAGCTGGG-TC**TGTAATCCCCATCACTGCAC** (letters in bold refer to the FXR cDNA) and after generation of an Entry Clone (in pDONR201, BP-Reaction, Invitrogen) recombined into pTRex-Dest30 (LR-reaction, Invitrogen) to yield pTRex-Dest30-hFXRα2, in which the coding region of human FXRα2 is under control of the CMVtetO2 promoter. For expression of FXR via an episomal vector, a 3751 bp SnaBI-SfiI fragment from pTReX-Dest30-hFXRα2 carrying the CMVteto2-hFXR-SV40polyA unit was inserted into the corresponding sites in pCEP4 (Life Technologies, Carlsbad, U.S.A.) to yield pCEP4-TReX-hFXRα2. To generate stable SK-Hep-1 and Huh7 clones (SK-GI-18 and Huh7-37) overexpressing human FXR, pTReX-Dest30-hFXRα2 was microporated into cells using microporation (Digital Bio Microporator MP-100, Peqlab, Erlangen, Germany) and stable clones selected in RPMI growth medium supplemented with 8.6% FCS and 1 mg/ml of G-418 (Life Technologies, Carlsbad, U.S.A.) and extracts of cells tested for overexpression of FXR in Western Blot using the mouse-anti-FXR antibody A9033A (R&D Systems, Wiesbaden-Nordenstadt, Germany). HepG2-FXR5 cells overexpressing human FXRα2 were generated by transfecting HepG2 cells with pCEP4-TReX-hFXRα2 and selecting a stable cell line in RPMI medium supplemented with 8.6% FCS and 200 µg/ml of Hygromycin B (Sigma-Aldrich, Munich, Germany).

The plasmid pMC3TRex is a derivative of pMEP4 (Invitrogen, Karlsruhe, Germany) in which the Metallothionine promoter was replaced by an expression cassette consisting of the E.coli tetracycline repressor controlled by the CMV promoter and the 3054 bp Sbf1-AscI fragment from pTReX-Dest30 which carries a gateway recombination cassette under control of the CMV-tetO2 promoter (Life Technologies, Carlsbad, U.S.A.). An N-terminal FLAG-tagged human FXR was cloned into pDONR201 and then recombined into pMC3TRex using a LR reaction (Life Technologies, Carlsbad, U.S.A.) to yield pMC3TRex-FLAG-FXR. Stable HepG2 cells carrying episomal pMC3TRex or pMC3TRex-FLAG-FXR were generated using microporation (Digital Bio Microporator MP-100, Peqlab, Erlangen, Germany) and selection of stable clones was done in RPMI growth medium supplemented with 8.6% FCS and 200 µg/ml of Hygromycin B (Life Technologies, Carlsbad, U.S.A.).

### Knock-down of NDRG2 in SK-GI-18 cells

For knock-down of NDRG2 expression, SureSilencing shRNA plasmids were purchased from Qiagen, Hilden Germany with the insert sequences 1: CAACCTGGATAACATTGAATT; 2: GGACCAGCTTGCAGACATGAT, 3: ACCAGTGCAGCATCCGTTCAT, 4: ATGTGGGACTCAACTATAAAT and as negative control NC: GGAATCTCATTCGATGCATAC under control of the U1 promoter and a hygromycin resistance marker gene. Stable SK-GI-18 cells were generated using microporation (Digital Bio Microporator MP-100, Peqlab, Erlangen, Germany) and stable clones selected in RPMI growth medium supplemented with 8.6% FCS and 200 µg/ml of Hygromycin B (Life Technologies, Carlsbad, U.S.A.) followed by growth in medium containing 1 mg/ml of G418 (Life Technologies, Carlsbad, U.S.A.).

### Protein detection by Western Blotting

Total protein was prepared from cells growing in 6 well tissue plates (Corning) by scraping using RLN buffer (50 mM Tris/Cl pH 8, 140 mM NaCl, 1.5 mM MgCl2, 0.5% (v/v) Nonidet P-40). Protease and Phosphatase inhibitors (Halt Protease Inhibitor Cocktail, Fischer Scientific GmbH, Schwerte, Germany, catalog# 78430) at a concentration of 1∶100 were freshly added to the RLN buffer before use.

Total cell lysates made in RLN buffer (10 µg) were separated by electrophoresis on 12% SDS-PAA gels, then electroblotted to Protran membranes (Whatman) in Tris transfer buffer containing 20% methanol. Membranes were stained with Ponceau S to verify loading and transfer efficiency. Membranes were probed with primary and secondary antibodies in Tris-buffered saline with Tween-20 (Sigma-Aldrich, Munich, Germany; TBS-T) containing 5% Soybean Hydrolysate (MP Biomedicals, Illkirch, France). Signals were visualized by incubating the blots in Lumi-Phos WB Chemiluminescent Substrate (Pierce, Rockford, USA) and exposing to X-ray film (MidSci, St. Louis, USA). Membranes were reprobed with anti-GAPDH antibodies after stripping with 0.2M Glycine for 2 hours.

### Gene expression analysis

Frozen tissues were ground to fine powder under liquid nitrogen with a mortar. Total RNA from 25 mg powdered tissue was isolated using RNAzol RT Reagent (MRC Inc., Cincinnati, U.S.A.) and an RNA Isolation Kit (RNeasy 96, Qiagen, Hilden, Germany) following instructions provided by the manufacturers. The concentration and integrity of the total RNA was assessed by gel electrophoresis and cDNA's were synthesized from 0.2 µg of total RNA using SuperscriptII™ reverse transcriptase (Life Technologies, Carlsbad, U.S.A.) primed with 50 pmol of random nonamers. Total RNA from cells grown in 96 well tissue plates were isolated following the instructions of the RNeasy 96 RNA isolation kit (Qiagen, Hilde, Germany) and cDNA's were synthesized from 1/25^th^ of the isolated total RNA from one 96 well. Quantitative PCR was performed and analyzed using Absolute QPCR Rox Mix (Life Technologies, Carlsbad, U.S.A.) and a 384-format ABI 7900HT Sequence Detection System (Applied Biosystems, Darmstadt, Germany). Primer and probe sequences (purchased from IDT DNA Technologies, Leuven, Belgium) are listed in supporting table S1. All samples were run at least in duplicates. Gene expression was expressed in arbitrary units and normalized relative to the housekeeping gene TATA box binding protein (TBP) from mouse or human, respectively.

FXR (NR1H4), NDRG2 and small heterodimer partner (SHP, NR0B2) mRNA levels were quantified in human normal liver, HCC and non-malignant liver disease cDNA's purchased from Origene (Cat# LVRT501, Rockville, USA). These cDNA samples have been generated from a total of 34 HCC, 8 normal livers and 12 non-HCC liver disease livers verified by pathologists before isolation of RNA and conversion to cDNA. The HCC samples included seven samples of stage I HCC, eight samples of stage II and IIIA HCC each, and three samples of stage IV HCC and 13 samples of non HCC liver diseases (http://www.origene.com/assets/documents/TissueScan/LVRT101.xls;). To study FXR, NDRG2, SHP and TBP expression, PrimeTime qPCR assays (IDT DNA Technologies, Leuven, Belgium) were used using the 384-format ABI 7900HT Sequence Detection System (Applied Biosystems, Darmstadt, Germany). FXR, BSEP, and SHP gene expression was normalized to TBP gene expression. The PrimeTime assays used in this study can be found in supporting table S1.

### Luciferase reporter assays

The IR1-type FXR response element from the human IBABP gene was cloned as a 68 bp KpnI-BglII fragment containing two IR1 elements (shown below) into the KpnI-BglII site of pGL2-promoter (Promega, Madison, U.S.A.) resulting in pGL2-IBABPx2.

5′Cccca**GGGTGAaTAACCT**cggggctctgtccctccaatccca**GGGTGAaTAACCT**cgggA

CATGGgggt**CCCACTtATTGGA**gccccgagacagggaggttagggt**CCCACTtATTGGA**gcccTCTAG 5 ′

The NDRG2 IR1-type response element shown below was cloned as a 66 bp Asp718-XhoI fragment into the Asp718 and XhoI sites of pGL4.30 (Promega, Madison, U.S.A.) replacing the NFAT response element resulting in pGL4-NDRG2-RE.

GTACCACACTGGGGA**GGGTTaGTGACC**CGAGG**AGGTTGaTGACCC**TGGGAGTCTGGGTCTTGGCTC

GTGTGACCCCT**CCCAATCACTGG**GCTCC**TCCAACtACTGGG**ACCCTCAGACCCAGAACCGAGAGCT

The NDRG2 IR1mut sequence shown below was cloned as a 66 bp Asp718-XhoI fragment into the Asp718 and XhoI sites of pGL4.30 (Promega, Madison, U.S.A.) replacing the NFAT response element resulting in pGL4-NDRG2mut.

GTACCACACTGGGGACCC**TTAGTGA**GGGGAGG**AGGTTGaTGA**GGGTGGGAGTCTGGGTCTTGGCTC

GTGTGACCCCTGGG**AATCACT**CCCCTCC**TCCAACtACT**CCCACCCTCAGACCCAGAACCGAGAGCT

The reporter plasmids (50 ng/well, pGL2-IBABPx2, pGL4-NDRG2 or pGL4-NDRG2mut) were cotransfected with pTRex-Dest30-hFXR (50 ng/well) and pCMV-RL (1 ng/well, constitutively expressing the *Renilla* luciferase under control of a CMV promotor as an internal control) into HEK293 cells and cultivated in presence of the FXR agonist Px20350 (0.5 µM) of DMSO for 16 h followed by cell lysis with Passive Lysis Buffer (Promega). Firefly and *Renilla* luciferase activities were measured sequentially in the same cell extract using a Dual-Light-Luciferase-Assay system and microplate luminometer (LUMIStar OPTIMA, BMG Labtech). Relative luciferase activity (RLA) was calculated as ratio between firefly luciferase and *Renilla* luciferase activity and relative fold induction calculated a RLA in presence of Px20350 divided by RLA in DMSO from at least three individual experiments.

#### Homogenous time-resolved fluoresecent resonance energy transfer assays (FRET) and Gal4-hybrid cellular reporter assays for mouse and human FXR

To assess the FXR agonist activity by TR-FRET a biotinylated SRC-1 peptide b-CPSSHSSLTERHKILHRLLQEGSPS-COOH (0.4 µM), 200 ng streptavidine-allophyco-cyanine and 6 ng europium labeled-anti-GST were mixed with either purified human FXR^aa187–472^ fused to GST, mouse FXR^aa202–484^ fused to GST (2.5 ng) in 25 µL assay buffer (20 mM Tris/HCl pH 7.5; 5 mM MgCl_2_; 60 mM KCl; 1 mM DTT; 0.9 g/L BSA). After 60 min of incubation the FRET efficiency was determined using a spectro-fluorometer (Envision, Perkin Elmer, Boston, U.S.A.). Measurements were done in duplicates. FRET values are given in nM and Eff (%) is the maximum efficacy of the compound relative to the control FXR agonist GW4064.

As cellular assay for FXR agonist activity, a Gal4-FXR fusion construct reporter system was used. HEK293 cells were transiently transfected with a reporter plasmid pFRluc (Stratagene, La Jolla, U.S.A.) encoding a Gal4 promotor driven firefly luciferase and a given FXR-LBD (human FXR^aa187–472^, mouse FXR^aa202–484^) expression plasmid fused C-terminally to a Gal4 DNA-binding domain under transcriptional control of the CMV promoter in pCMV-BD (Stratagene La Jolla, U.S.A.). Additionally a second reporter plasmid pRL-TK constitutively expressing the *Renilla* luciferase under control of a TK promotor was included as an internal control. 4 h after transfection cells were treated with serial dilutions of the test FXR agonists. 16 h after treatment cells were lysed with Passive Lysis Buffer (Promega, Madison, U.S.A.) and firefly and *Renilla* luciferase activities were measured sequentially in the same cell extract using a Dual-Light-Luciferase-Assay system and microplate luminometer (LUMIStar OPTIMA, BMG Labtech, Ortenberg, Germany). Relative luciferase activity was calculated and reported as ratio between firefly luciferase and *Renilla* luciferase activity. EC_50_ values were calculated from at least three independent experiments.

### Chromatin Immunoprecipitation

HepG2 cells stably transfected with pMC3TRex or pMC3TRex-FLAG-FXR were grown in 10 cm cell culture dishes to 70% confluency and cells fixed and chromatin sheared using the Shearing ChIP Kit and the Bioruptor (Diagenode, Liege, Belgium). For chromatin immunoprecipitation, the One-day ChIP kit was used according to the recommended protocol (Diagenode, Liege, Belgium). One 20^th^ of the sheared material was used for isolation of input DNA and aliquots of sheared chromatin corresponding to 1×10^6^ cells was incubated with 5 µg affinity purified M2 anti-FLAG antibody (F1804, Sigma-Aldrich, Munich, Germany) in 300 µl ChIP buffer with mild sonication (Diagenode Bioruptor, medium setting, 15 min and a 0.5 min on-off-cycle). PCR was done with forward GAACTGATGCCCTTGTAGCC and reverse CAACGAGGTGAATGACATGG primers, for 41 cycles (15 sec 95°C and 60 sec 60°C) to amplify a 196 bp DNA fragment harbouring the IR1 sequence.

### Intrahepatic injection

Mice were anesthetized with isoflurane and 5×10^6^ SK-Hep-1 or 5×10^6^ SK-GI-18 cells were resuspended in a volume of 100 µl PBS and injected into one liver lobe, respectively, according to the SOP established at Oncotest Freiburg, Germany.

### *In vivo* imaging

Mice were injected intravenously with 0.02 mg anti-human-CD10-Antibody (AbD Serotec, Düsseldorf, Germany), that is labelled with fluorescence dye Xenolight CF750 (Caliper Life Sciences, Mainz, Germany). Mice were anesthetised by isoflurane inhalation 20 hours after the injection, and pictures (fluorescence and x-ray) were taken with a Kodak in vivo imaging system (Kodak Image Station in vivo FX).

### Statistical analysis

Data were expressed as the means ± SEM from at least three experimental measurements.

Differences between experimental groups were evaluated for statistical significance using Student's t-test: *, p<0.05; **, p<0.01; ***, p<0.001. using the software Prism5 (GraphPad Software Inc., San Diego, USA). where * p<0.05 is considered to be statistically significant.

## Supporting Information

Figure S1 **Small hairpin mediated knock-down of NDRG2 mRNA in SK-GI-18-sh3-14 cells compared to SK-GI-18-NC16 cells.** Cells were grown in triplicates in 6 well plates in medium containing DMSO (D) or 1 µM PX20606 (PX) in DMSO for 18 h before the relative NDRG2 mRNA levels were determined by RT-qPCR from isolated total RNA. The NDRG2 mRNA levels were normalized to TBP and the relative NDRG2 mRNA levels in SK-GI-18-NC16 cells grown with DMSO set to 1 and Students ttest performed.
(DOCX)

Table S1 **Sequences of primers for PrimeTime-Assays (qPCR) supplied by IDT DNA Technologies.**
(DOCX)
