# Supplementary material for: FXR Controls the Tumor Suppressor NDRG2 and FXR Agonists Reduce Liver Tumor Growth and Metastasis in an Orthotopic Mouse Xenograft Model
Source: PLoS One. 2012 Oct 9;7(10):e43044. doi: 10.1371/journal.pone.0043044 (PMC3467263; doi:10.1371/journal.pone.0043044)

Supporting Figure S1

Small hairpin mediated knock-down of NDRG2 mRNA in SK-GI-18-sh3-14 cells compared to SK-GI-18-NC16 cells. Cells were grown in triplicates in 6 well plates in medium containing DMSO (D) or 1µM PX20606 (PX) in DMSO for 18h before the relative NDRG2 mRNA levels were determined by RT-qPCR from isolated total RNA. The NDRG2 mRNA levels were normalized to TBP and the relative NDRG2 mRNA levels in SK-GI-18-NC16 cells grown with DMSO set to 1.0 and Students ttest performed.


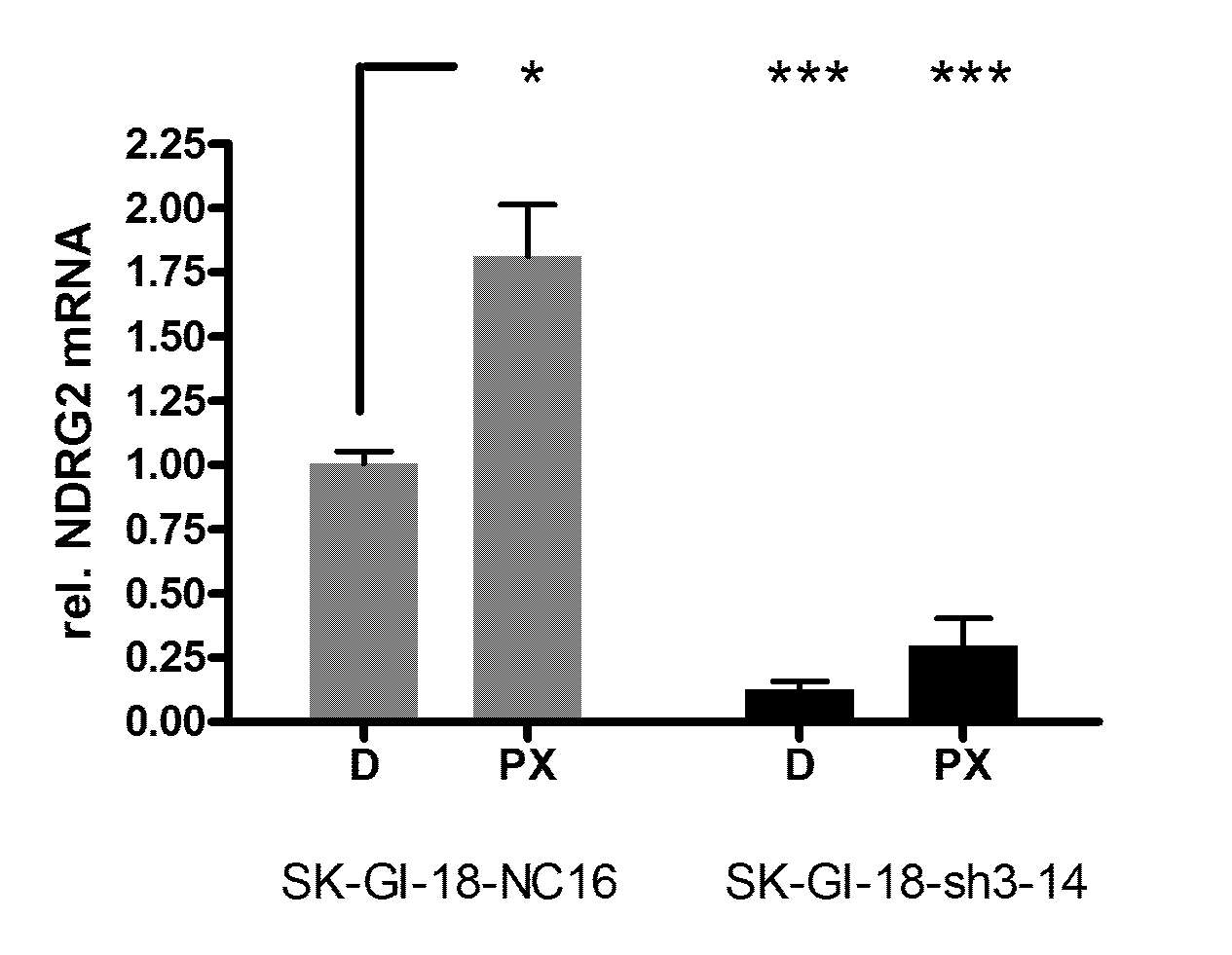

Supplement: Figure S1 — Small hairpin mediated knock-down of NDRG2 mRNA in SK-GI-18-sh3-14 cells compared to SK-GI-18-NC16 cells. Cells were grown in triplicates in 6 well plates in medium containing DMSO (D) or 1 µM PX20606 (PX) in DMSO for 18 h before the relative NDRG2 mRNA levels were determined by RT-qPCR from isolated total RNA. The NDRG2 mRNA levels were normalized to TBP and the relative NDRG2 mRNA levels in SK-GI-18-NC16 cells grown with DMSO set to 1 and Students ttest performed. (DOCX) [file pone.0043044.s001.docx]
