# Supplementary material for: FXR Controls the Tumor Suppressor NDRG2 and FXR Agonists Reduce Liver Tumor Growth and Metastasis in an Orthotopic Mouse Xenograft Model
Source: PLoS One. 2012 Oct 9;7(10):e43044. doi: 10.1371/journal.pone.0043044 (PMC3467263; doi:10.1371/journal.pone.0043044)
Supplement: Table S1 — Sequences of primers for PrimeTime-Assays (qPCR) supplied by IDT DNA Technologies. (DOCX) [file pone.0043044.s002.docx]

**Supporting Table S1:** Sequences of primers for PrimeTime-Assays supplied by IDT DNA Technologies

| **Gene** | **species** | **Refseq** | **Forward Primer** | **Reverse Primer** | **Sequence Probe** |
| --- | --- | --- | --- | --- | --- |
| Tbp | mouse | NM_013684 | AAGAAAGGGAGAATCATGGACC | GAGTAAGTCCTGTGCCGTAAG | CCTGAGCATA AGGTGGAAGGCTGTT |
| Fxr-a | mouse | NM_009108 | GGACGGGATGAGTGTGAAG | TGAACTTGAGGAAACGGGAC | AGGTATGCTAACAGAACACGCGGC |
| Ndrg2 | mouse | NM_013864 | TGAACTTTGAGCGAGGTGG | CATCTTGAGGAACGAGGTCTG | ATCACAGGGCACTTGAGGGTCG |
| Shp | mouse | NM_011850 | CTACCCTCAAGAACATTCCAGG | CACCAGACTCCATTCCACG | CAGTGATGTCAACGTCTCCCATGATAGG |
| Cyp7a1 | mouse | NM_007824 | AACGATACACTCTCCACCTTTG | CTGCTTTCATTGCTTCAGGG | TGTTTGCTTGAGATGCCCAGAGGA |
| Cyclophilin E | mouse | NM_019489 | GCTGCATTTATCCCCTTTGG | ATCCTCTGCCAACTCAAACTC | TGAAACAGAAAAACACCGAGGGTTTGC |
| TBP | human | NM_003194 | TTCTGGGATTGTACCGCA | AGCAAACCGCTTGGGA | CGTGCCCGAAACGCCGAATAT |
| FXR | human | NM_005123 | AGCTGAACGAAGGA CAT | GACGGAAATGGCAACCAA | ACTTTGGACCATGAAGACCAGATTGCT |
| NDRG2 | human | NM_201535 | TTTGAGCGTGGAGGTGATATC | CATCTTGAGGAACGAGGTCTG | CATCTTCATGAGGTGCTTGGTCTCCTAC |
| SHP | human | NM_021969 | TTAGCCCCAAGGAATATGCC | GGGTTCCAGGACTTCACAC | TC CTC TTC AA CCC CGATGTGCC |
